# Reliability of Resting Heart Rate-based Target Heart Rate for Exercise Prescription after Acute Myocardial Infarction

**DOI:** 10.1298/ptr.25-E10371

**Published:** 2025-11-22

**Authors:** Yuya UTSUMI, Koji TAKASE, Naoya MURAKAMI, Tokiko NAKAGAWA, Takuya OBAYASHI, Riyo OGURA, Shinobu HOSOKAWA

**Affiliations:** 1 Department of Rehabilitation, Tokushima Red Cross Hospital, Japan; 2 Department of Cardiology, Tokushima Red Cross Hospital, Japan

**Keywords:** Target heart rate, Resting heart rate, Anaerobic threshold, Acute myocardial infarction, Hemoglobin

## Abstract

**Objectives:**

Anaerobic threshold (AT) assessment using cardiopulmonary exercise testing (CPX) during hospitalization is considered ideal for prescribing exercise regimens in patients with acute myocardial infarction (AMI). However, practical limitations often hinder its implementation. This study evaluated strategies for prescribing exercise based on resting heart rate (RHR).

**Methods:**

A total of 194 consecutive male patients with AMI who underwent CPX within 13 days of hospitalization were enrolled. Bland–Altman analysis was performed to assess the agreement between the heart rate (HR) at AT (ATHR) and RHR +20, +25, and +30 beats. Multiple regression analysis was conducted to identify factors influencing ΔHR (ATHR−RHR). Decision-tree analysis was used to establish thresholds for appropriate exercise prescriptions based on RHR.

**Results:**

Regardless of β-blocker use, the RHR + 25 formula most closely approximated the ATHR in patients who underwent early CPX after AMI. Multivariate analysis identified RHR and hemoglobin (Hb) as significant predictors of ΔHR. Decision-tree analysis indicated that RHR + 25 was appropriate when RHR <91 bpm and Hb ≥12.0 g/dL.

**Conclusions:**

RHR + 25 is a practical alternative for determining the target HR in post-AMI rehabilitation among male patients, particularly in those with RHR <91 bpm and Hb ≥12.0 g/dL, when CPX is not feasible.

## Introduction

Cardiac rehabilitation (CR) is an evidence-based intervention that reduces mortality and rehospitalization rates while improving quality of life in patients with cardiovascular diseases such as acute coronary syndrome^1)^. Methods to determine the target heart rate (HR) during exercise include the Karvonen method, which involves 40%–60% of the HR reserve, the resting HR (RHR) + 20–30 beats, and anaerobic threshold (AT) assessment using cardiopulmonary exercise testing (CPX)^2)^.

Guidelines for the rehabilitation of patients with cardiovascular disease indicate that symptom-limited CPX should be performed 14 days after the onset of acute myocardial infarction (AMI)^2)^. Recently, the duration of hospital stay after percutaneous coronary intervention in patients with AMI has shortened significantly^3)^. Therefore, determining the target HR using the Karvonen method and HR reserve, both of which involve maximal HR assessment, is difficult during hospitalization. The widely used “220 − age” formula for estimating maximal HR is simple but lacks accuracy, particularly for individualized exercise prescription. Major professional organizations such as the American College of Sports Medicine and the American Council on Exercise have indicated that this equation tends to overestimate maximal HR in younger individuals and underestimate it in older adults, with a standard deviation of ±10–15 bpm^4,5)^. Due to this considerable variability, they recommend caution when applying the formula in clinical or rehabilitation settings.

Assessing AT using CPX is recommended for determining individualized exercise intensity, as well as for appropriate exercise prescriptions. Reports from Japan have also indicated that inappropriate exercise prescriptions not based on the results of individualized exercise testing are highly likely to cause adverse events^6)^. However, it is considered feasible to perform an evaluation up to the AT between 4 and 6 days after onset^2)^. Although exercise at the AT is considered safe and beneficial, exceeding this intensity may pose risks; therefore, CR based on individualized exercise prescriptions is recommended^6–10)^. However, owing to patient or equipment limitations, CPX may not always be feasible, necessitating the accurate prediction of the AT using the RHR for exercise prescriptions. In such cases, arbitrary methods for prescribing exercise intensity, such as RHR + 20/+30 bpm, are recommended for inpatients with AMI after the recovery phase^2)^. In a national survey regarding exercise prescriptions in the United States involving cardiac rehabilitation program directors, the most standard method for prescribing exercise intensity was the rating of perceived exertion, followed by RHR + 20–30^11)^. Exercise prescription using RHR + 20–30 may not accurately approximate the HR at the AT (ATHR), which is considered the gold standard for target HR determination using CPX.

However, the characteristics of these cases have not been thoroughly investigated. In Japan, where the implementation rate of outpatient CR is low^12,13)^, administering accurate exercise prescriptions within a short hospitalization period and developing exercise prescriptions for home-based CR are crucial. Accordingly, AT evaluation during shorter hospitalization periods and CR implementation based on individualized exercise prescriptions are important to ensure the safe continuation of home-based exercise therapy after the acute phase.

Therefore, this study aimed to evaluate the reliability of exercise prescriptions based on the RHR, identify the characteristics of cases where it can be appropriately applied, and establish a strategy for determining the target HR using the RHR.

## Methods

### Participants

**Fig. 1.**
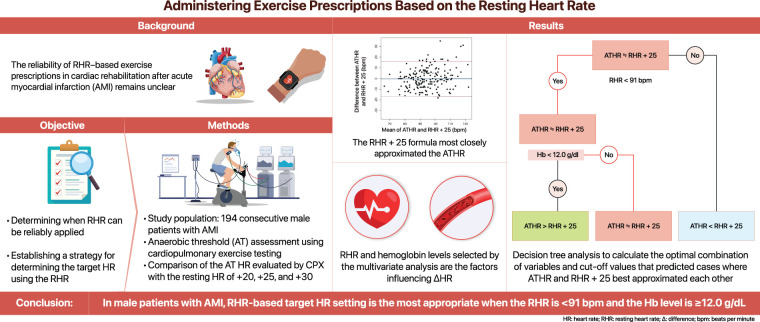
This study evaluated strategies for exercise prescription based on RHR after AMI. AMI, acute myocardial infarction; ΔHR, difference between the ATHR and RHR; ATHR, heart rate at anaerobic threshold; CPX, cardiopulmonary exercise testing; RHR, resting heart rate

**Fig. 2.**
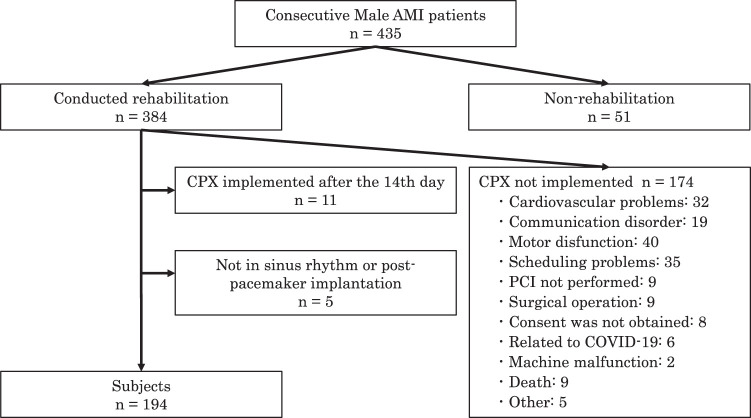
Flowchart of the study population. AMI, acute myocardial infarction; COVID-19, coronavirus disease 2019; CPX, cardiopulmonary exercise testing; PCI, percutaneous coronary intervention

Male patients who presented to our hospital with AMI between July 2021 and 2024 were included (Fig. 1). The exclusion criteria were: no opportunity to undergo rehabilitation, inability to undergo CPX, CPX administered after day 14 of hospitalization, and not being in sinus rhythm. Refer to Figure 2 for details on the excluded participants. The data used in this study were obtained from an ongoing prospective cohort study (Approval No. 758), approved by the Ethics Committee of Tokushima Red Cross Hospital. All participants provided written informed consent. All procedures involving human participants were conducted following the ethical standards of the institutional review board and the Declaration of Helsinki.

### Assessments

#### CPX

For CPX measurements, an exercise stress electrocardiogram device (STS-2100; Nihon Kohden, Tokyo, Japan), ergometer (Strength Ergo 8 BK-ERG121; Fukuda Denshi, Tokyo, Japan), and expiratory gas metabolism monitor (CPEX-1; Inter-Reha, Tokyo, Japan) were used. The CPX protocol involved a 3-min seated rest, followed by a 3-min warm-up and ramp load at 10 W each; the test concluded after AT evaluation. The AT was determined using the V-slope method or the widely used ventilatory equivalent method, as appropriate^14)^. The resting HR was defined as the average HR during the last 1 min of a 3-min resting seated period, measured while monitoring the electrocardiogram during CPX. The difference between the ATHR and RHR was defined as the ΔHR. The timing of CPX was not standardized; however, it was conducted while avoiding the period immediately after morning medication administration.

#### Muscle strength

Grip strength was measured using a Smedley-type digital grip strength meter (Digital Grip-D; Takei Kiki, Niigata, Japan). The measurement was performed once with the dominant hand, with the limb in a standing position.

Isometric knee extension strength was measured using a hand-held dynamometer (μTasF-100; Anima, Tokyo, Japan). The patient was seated on a bed with both upper limbs mildly abducted and the lower leg drooping. An isometric knee extension motion with maximal effort was performed once for approximately 5 seconds on the lower limb ipsilateral to the dominant hand. The value was divided by the body weight to account for knee extension muscle strength. The measurements were performed within 3 days before discharge.

#### Skeletal muscle mass index and phase angle

Skeletal muscle mass index (SMI) was measured using a multifrequency body composition analyzer (MC-780A-N; Tanita, Tokyo, Japan). Variables were measured according to a previously recommended method^15)^. SMI was calculated by dividing the amount of skeletal muscle in the four limbs by height squared, according to the method recommended by the Asian Working Group for Sarcopenia^16)^. In addition, the phase angle (PhA)—the ratio of resistance (resistance) between the cell’s outer and inner fluids and resistance (reactance) caused by the cell membrane at 50 kHz, expressed as an angle—was evaluated. The measurements were performed within 3 days before discharge.

#### Physical activity

The International Physical Activity Questionnaire (IPAQ) long form was used to evaluate the participants’ physical activity levels^17)^. Participants were asked to report the days and times they performed physical activity during the seven days prior to hospitalization. The data collected from the long IPAQ questionnaires were summed within each physical activity domain to estimate the total time spent in occupational, transport, household, and leisure-related physical activity, as well as the total time reported for sedentary behavior per week. The weighted metabolic equivalent (METs)-minutes per week (METs· min· wk-1) were calculated as duration × frequency per week × METs intensity, which were summed across activity domains to produce a weighted estimate of total physical activity from all reported activities per week (METs· min· wk-1)^17)^. For the sedentary behavior question, “minutes” is used as the indicator to reflect time spent in sedentary behavior, rather than METs-minutes, which would suggest an estimate of energy expenditure.

#### Other measures

The analysis included blood test results, Killip’s classification, and echocardiographic parameters. Blood tests comprised hemoglobin (Hb) and estimated glomerular filtration rate (eGFR), which were obtained from the test performed closest to the CPX, and HbA1c, which was measured at the first blood sampling on admission. Peak creatine kinase (CK) was defined as the highest value regardless of timing. Killip’s classification was used to assess the severity of AMI. Echocardiographic parameters included left ventricular ejection fraction (LVEF) and left atrial diameter (LAD), which were measured once during hospitalization after transfer from the intensive care unit to the general ward; the timing was not standardized and did not occur in the ultra-acute phase.

### Contents of rehabilitation

During hospitalization, patients underwent rehabilitation in accordance with the AMI rehabilitation program prescribed by the attending physician. Our rehabilitation program was based on the program listed in the rehabilitation guidelines for patients with cardiovascular diseases^2)^.

### Statistical analysis

Normally distributed continuous variables are expressed as mean ± standard deviation, while those with skewed distributions are expressed as median (interquartile range). Categorical variables are expressed as numbers (percentages).

The consistency between ATHR and RHR + 20, +25, and +30 was examined using Bland–Altman analysis, respectively. The same analysis was also performed after categorizing the subjects based on the presence or absence of β-blockers (with β-blockers: n = 134, 69.1%).

We investigated the factors influencing the ΔHR, defined as the difference between the ATHR and RHR. We used multiple regression analysis with ΔHR as the dependent variable. Prior to the analysis, a lasso regression was performed with ΔHR as the dependent variable, and variables potentially associated with ΔHR were selected based on the optimal log (lambda) value. The independent variables included age, BMI, LVEF, LAD, Hb, HbA1c, eGFR, peak CK, RHR, grip strength, knee extension muscle strength, SMI, PhA, total physical activity level, leisure-time activity level, and average sedentary behavior time.

Additionally, a decision-tree analysis was conducted to calculate the optimal combination of variables and cut-off values for predicting cases in which ATHR and RHR + 25 approximate each other. For all subjects, cases with a difference between ATHR and RHR + 25 within ± 5 were classified as ATHR ≒ RHR + 25 (n = 87, 44.9%), those with a difference of ≥ +6 as ATHR >RHR + 25 (n = 46, 23.7%), and those with a difference of ≤6 as ATHR <RHR + 25 (n = 61, 31.4%). The existence of several nodes before reaching a leaf in a decision tree presents an increased risk of overlearning; therefore, it is desirable to reach a leaf with as few variables as possible. In this study, since the results indicated that overlearning was least likely to occur when the size of the tree was 2, trees and nodes beyond the third are omitted in the description. Independent variables for this analysis were selected from the previous multiple regression analysis.

All analyses were performed using the modified R Commander (version 4.2.1 for macOS; Freeware, CRAN, Vienna, Austria). Statistical significance was set at p <0.05.

## Results

### Patient characteristics

A flowchart of the patient selection process is shown in Figure 2. Initially, 435 men with AMI were assessed for eligibility. After applying the exclusion criteria, 194 individuals were included in the subsequent analyses. Tables 1 and 2 summarize the patient characteristics and physical function, respectively. The mean age of the participants was 65.5 (54.0–74.0) years, with a BMI of 24.9 (22.6–27.5) kg/m^2^.

**Table 1.** Patient’s characteristics

	All subjects (n = 194)
Basic information	
Age (years)	65.5 (54.0–74.0)
BMI (kg/m^2^)	24.9 (22.6–27.5)
Emergency PCI (n)	188 (96.9)
Pre-emergency PCI (n)	6 (3.1)
Length of hospital (day)	8 (7–10)
Killip’s classification (n)	I: 165 (85.1)
	II: 22 (11.3)
	III: 2 (1.0)
	IV: 5 (2.6)
IABP used (n)	25 (12.9)
Adaptation program (n)	1 week: 137 (70.6)
	10 days: 52 (26.8)
	Other: 5 (2.6)
Medical history	
Hypertension (n)	135 (69.6)
Diabetes mellitus (n)	67 (34.5)
Hyperlipidemia (n)	120 (61.9)
Currently smoking (n)	82 (42.3)
Medication	
Antiplatelet drug (n)	194 (100)
Renin–angiotensin–aldosterone system inhibitor (n)	167 (86.1)
β-blocker (n)	134 (69.1)
Sodium glucose transporter 2 inhibitor (n)	52 (26.8)
HMG-CoA reductase inhibitors (n)	192 (99.0)
Various inspection results	
LVEF (%)	57.0 (51.0–62.0)
LAD (mm)	42.0 (39.3–46.0)
Peak CK (U/L)	2215.0 (1051.0–3378.8)
Hemoglobin (g/dL)	13.3 (12.4–14.3)
Hemoglobin A1c (%)	6.0 (5.7–6.9)
eGFR (mL/min/1.73 m^2^)	63.5 ± 15.6
STEMI (n)	184 (94.9)
Diseased branch (n)	RCA: 75 (38.9)
	LMT: 4 (2.1)
	LADA: 80 (41.5)
	LCX: 31 (16.1)
	HL: 3 (1.6)
Remnant branch (n)	102 (52.6)

Variables are expressed as mean ± SD, median (IQR), or number of patients (%).

BMI, body mass index; PCI, percutaneous coronary intervention; IABP, intra-aortic balloon pumping; LVEF, left ventricular ejection fraction; LAD, left atrial diameter; peak CK, peak creatine kinase; eGFR, estimated glomerular filtration rate; STEMI, ST-elevation myocardial infarction; RCA, right coronary artery; LMT, left main coronary trunk artery; LADA, left anterior descending artery; LCX, left circumflex artery; HL, high lateral branch; SD, standard deviation; IQR, interquartile range

**Table 2.** Physical functioning of patients

	All subjects (n = 194)
CPX	
Implemented (hospital day)	7 (6–8)
AT (mL/min/kg)	12.0 (10.5–13.7)
WR at AT (W)	52.0 (44.0–58.0)
${\dot{V}}_{\text{E}}$ vs ${\dot{V}}_{{\text{CO}}_{2}}$ slope	27.7 (23.9–33.6)
RHR (bpm)	69.6 ± 9.5
ATHR (bpm)	94.3 ± 12.7
ΔHR (bpm)	24.0 (17.0−30.0)
Association between ATHR and RHR + 25 (n)	ATHR >RHR + 25: 46 (23.7)
	ATHR≒ RHR + 25: 87 (44.9)
	ATHR<RHR + 25: 61 (31.4)
Skeletal muscle function	
Assessment (hospital day)	6 (5−7)
Grip strength (kg)	34.4 ± 7.8
Knee extension per weight (%)	41.3 ± 10.4
SMI (kg/m^2^)	8.0 ± 1.0
PhA (°)	5.8 ± 0.8
Physical activity	
Total physical activity (METs· min· wk-1)	980.5 (198.5–2182.5)
Leisure time physical activity (METs· min· wk-1)	0 (0–720.0)
Sedentary behavior (min/day)	270.0 (150.0–417.9)

Variables are expressed as mean ± standard deviation or median (interquartile range).

CPX, cardiopulmonary exercise testing; AT, anaerobic threshold; WR, work rate; RHR, resting heart rate; ATHR, heart rate the AT; ΔHR, difference between the ATHR and RHR; SMI, skeletal muscle mass index; PhA, phase angle; MET, metabolic equivalent

### Bland–Altman analysis

**Fig. 3.**
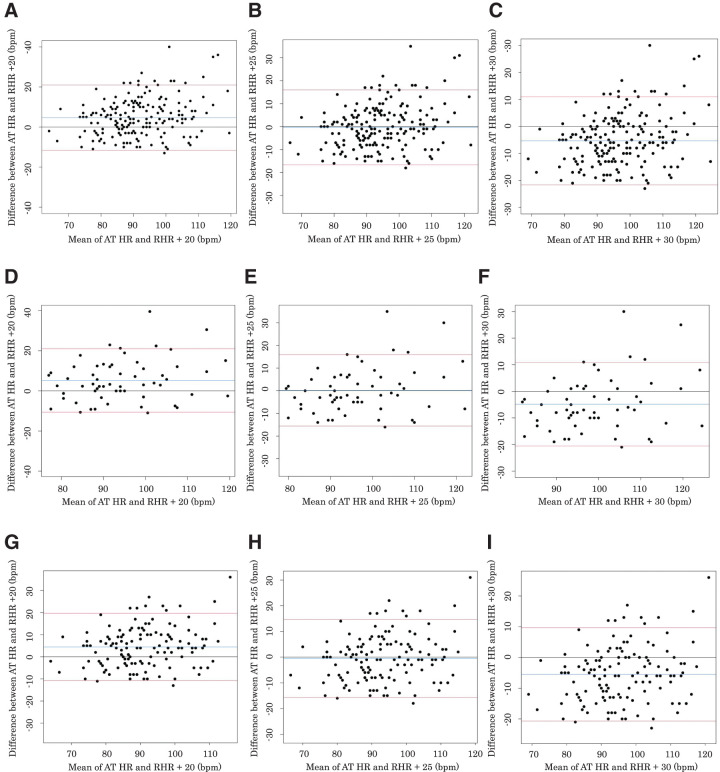
Bland–Altman analysis results for all subjects, subjects not prescribed β-blockers, and subjects prescribed β-blockers. (A) Comparison between the ATHR and RHR + 20 for all subjects. (B) Comparison between the ATHR and RHR + 25 for all subjects. (C) Comparison between the ATHR and RHR + 30 for all subjects. (D) Comparison between the ATHR and RHR + 20 for subjects not prescribed β-blockers. (E) Comparison between the ATHR and RHR + 25 for subjects not prescribed β-blockers. (F) Comparison between the ATHR and RHR + 30 for subjects not prescribed β-blockers. (G) Comparison between the ATHR and RHR + 20 for subjects prescribed β-blockers. (H) Comparison between the ATHR and RHR + 25 for subjects prescribed β-blockers. (I) Comparison between the ATHR and RHR + 30 for subjects prescribed β-blockers. ATHR, heart rate at the anaerobic threshold; RHR, resting heart rate

The results of the Bland–Altman analysis for all subjects, those not prescribed β-blockers (n = 60), and those prescribed β-blockers (n = 134) are shown in Figure 3. To begin with, the results for all subjects are presented in Figures 3A–3C. The consistency analysis for ATHR and RHR + 20 revealed that both fixed and proportional errors were significant (both p <0.05; Fig. 3A). For ATHR and RHR + 25, fixed errors were not significant (p = 0.64), whereas proportional errors were significant (p <0.05; Fig. 3B). The consistency analysis for ATHR and RHR + 30 showed that both fixed and proportional errors were significant (both p <0.05; Fig. 3C). The results of the Bland–Altman analysis for subjects not prescribed β-blockers are shown in Figures 3D–3F (n = 60). The consistency analysis for ATHR and RHR + 20 showed that both fixed and proportional errors were significant (both p <0.05; Fig. 3D). For ATHR and RHR + 25, fixed errors were not significant (p = 0.92), whereas proportional errors were significant (p <0.05; Fig. 3E). The consistency analysis for ATHR and RHR + 30 showed that both fixed and proportional errors were significant (both p <0.05; Fig. 3F). The results of the Bland–Altman analysis for subjects prescribed β-blockers are shown in Figures 3G–3I (n = 134). The consistency analysis for ATHR and RHR + 20 showed that both fixed and proportional errors were significant (both p <0.05; Fig. 3G). For ATHR and RHR + 25, fixed errors were not significant (p = 0.51), whereas proportional errors were significant (p <0.05; Fig. 3H). The consistency analysis for ATHR and RHR + 30 showed that both fixed and proportional errors were significant (both p <0.05; Fig. 3I).

### Multivariate analysis

Table 3 shows the results of the multiple regression analysis for predictors of the ΔHR. Among the independent variables analyzed, both RHR and Hb were identified as significant factors (multiple R^2^: 0.09; adjusted R^2^: 0.08). The regression coefficients for RHR and Hb were β = −0.16 (95% confidence interval [CI]: −0.28 to −0.04; p <0.05) and β = −1.28 (95% CI: −2.12 to −0.43; p <0.05), respectively. The independent variables (RHR, Hb level, BMI, LAD, grip strength, and knee extension strength) were selected through a prior lasso regression. Age was forcibly included as an independent variable in the multivariable analysis regardless of the results of the lasso regression.

**Table 3.** Multiple regression analysis for predictors of ΔHR

	β	95% CI for β	Standard partial regression coefficient	p Value
RHR (bpm)	−0.16	−0.28, −0.04	−0.18	<0.05
Hemoglobin (g/dL)	−1.28	−2.12, −0.43	−0.21	<0.05
Age (years)	—	—	—	—
BMI (kg/m^2^)	—	—	—	—
LAD (mm)	—	—	—	—
Grip strength (kg)	—	—	—	—
Knee extension strength (%)	—	—	—	—

Multiple R^2^: 0.09; adjusted R^2^: 0.08.

ΔHR, difference between the heart rate the anaerobic threshold and RHR; RHR, resting heart rate; BMI, body mass index; LAD, left atrial dimension; CI, confidence interval

### Decision-tree analysis

**Fig. 4.**
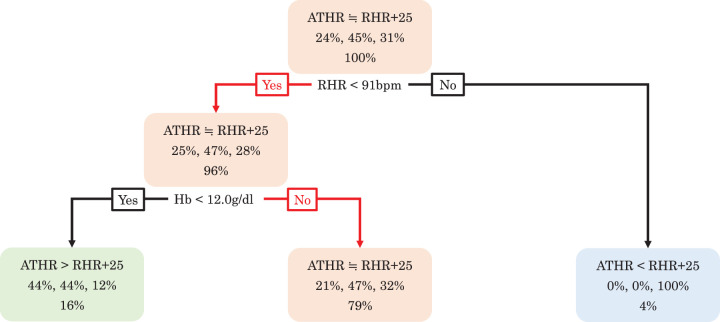
Results of the decision-tree analysis The results of the decision-tree analysis were used to calculate the optimal combination of variables and cutoff values that predicted cases where the ATHR and RHR + 25 best approximated each other. The value in the middle part of the node indicates the proportion of patients who meet the conditions from left to right: ATHR >RHR + 25, ATHR ≒ RHR + 25, and ATHR <RHR + 25, relative to the total number of patients in the node. The value in the lower part of the node is the ratio of the number of patients matching the node’s criteria to the total number of patients. ATHR, heart rate at the anaerobic threshold; Hb, hemoglobin; RHR, resting heart rate

The results of the decision-tree analysis are shown in Figure 4. Exercise prescription based on RHR + 25 was most appropriate when the RHR and Hb level were <91 bpm and ≥12.0 g/dL (red arrow in Fig. 4), respectively. For subjects with an RHR ≥91 bpm, an exercise prescription based on RHR + 25 was likely to exceed the ATHR, resulting in an increased risk of overload. Conversely, in subjects with an RHR <91 bpm and Hb <12.0 g/dL, setting RHR + 25 as the target HR may fail to reach the ATHR, potentially resulting in an insufficient exercise load.

## Discussion

Our study evaluated the reliability of exercise prescriptions based on the RHR in male patients with AMI who were in sinus rhythm, identified situations where this approach is appropriately applicable, and established a strategy for determining the target HR using the RHR. The results suggest that the RHR + 25 formula most closely approximates the ATHR, with consistent results regardless of β-blocker use. Multiple regression analysis—with ΔHR as the dependent variable—identified RHR and Hb level as significant predictors. Hence, individuals with a low RHR and Hb levels experienced a larger ΔHR from rest to AT. The decision-tree analysis results indicate that RHR-based target HR setting is most appropriate when the RHR and Hb levels are <91 bpm and ≥12.0 g/dL, respectively, and in situations where CPX cannot be performed. Compared with the Japanese Circulation Society (JCS) Guidelines, which define the upper limit of exercise intensity as <120 bpm or an increase of <40 bpm from the RHR, the RHR + 25 formula provides a more specific and clinically practical target^2)^. In patients with an RHR ≥91 bpm, even when the target HR is set at RHR + 25, it may lead to exercise overload and is therefore not appropriate for determining exercise intensity. In other words, in such cases, it is difficult to use an exercise prescription based on RHR, and it becomes necessary to provide guidance on methods for determining exercise intensity, such as the rate of perceived exertion and the talk test. In subjects with RHR <91 bpm and Hb <12.0 g/dL, setting the target HR at RHR + 25 may still fail to reach the ATHR, resulting in an insufficient load. Therefore, in such cases, it may be appropriate to use the guidelines as a reference and aim for an increase of 30–40 bpm from the RHR^2)^. This specificity may facilitate easier application in clinical settings, particularly during early rehabilitation after AMI. Consequently, our findings may offer a more practical option for exercise prescription that complements the current guidelines. Given the reduced hospitalization periods following the introduction of percutaneous coronary intervention^3)^ and the low implementation rate of outpatient CR in Japan^12,13)^, our findings in patients with sinus rhythm provide important insights for developing appropriate exercise prescriptions for home-based CR after discharge.

The HR response to exercise is regulated by the balance between sympathetic and parasympathetic nervous system activity^18)^. A lower RHR is associated with a better prognosis in patients with heart failure.^19,20)^ The suggested relationship between elevated baseline HR and increased mortality is deemed reasonable in clinical settings, as an elevated HR may reflect neurohumoral activation of the sympathetic nervous system. In this study, patients with more severe myocardial infarction (MI) may have exhibited an elevated RHR due to excessive sympathetic nervous system activation; as a result, the AT may have been reached earlier, resulting in a smaller ΔHR.

Hb levels were identified as a significant predictor of ΔHR in the multivariate analysis. This can be attributed to the fact that 1 g of Hb chemically binds to 1.34 mL of oxygen, facilitating its transport to the peripheral organs. Consequently, in individuals with lower Hb levels, a compensatory increase in HR is likely to occur to meet the oxygen demand of the peripheral tissues. Figure 4 shows a tendency for the ATHR to be higher than RHR + 25 when Hb is <12 g/dL. In cases where cardiac function is impaired due to MI, stroke volume cannot be sufficiently increased during incremental exercise, making the increase in cardiac output more dependent on the HR. This tendency was more pronounced in patients with lower Hb levels as a compensatory mechanism to transport oxygen to the peripheral organs.

It is widely accepted that age impacts cardiac reserve. In this study, we initially hypothesized that age would influence ΔHR and therefore forcibly included it as an independent variable in the multivariate analysis. However, age was not selected as a significant predictor. One possible explanation is the limited age range of our participants, who were predominantly middle-aged men (median 65.5 years; interquartile range 54.0–74.0; Table 1). If a broader age range from young to older adults had been included, age might have been selected. Moreover, the study specifically enrolled AMI patients who were able to perform a bicycle ergometer test, excluding those with sarcopenia or musculoskeletal disorders common in older adults, further narrowing the age distribution. Therefore, the lack of association between age and ΔHR in our analysis likely reflects the characteristics of the study population rather than indicating that age has no effect on ΔHR after AMI.

Regardless of the use of β-blockers, RHR + 25 most closely approximated the ATHR. Beyond the recovery phase for patients with AMI, guidelines recommend setting exercise intensity using the RHR, suggesting RHR + 20 and RHR + 30 for patients taking and not taking β-blockers, respectively; however, our findings contradict this recommendation.^2)^ A previous study investigated the effect of carvedilol on HR in patients with subacute MI and revealed no significant difference between the use and nonuse of carvedilol^21)^. In the multivariate analysis of factors affecting ΔHR, the use of β-blockers was not a significant variable. This may be because β-blockers decrease HR responses during moderate-to-high intensity exercise^22)^. In this study, the HR response from RHR to AT was examined, which may not have been influenced by the use of β-blockers.

In the examination of the agreement between ATHR and RHR + 25, a proportional bias in the positive direction was observed in all cases. This may be attributed to the presence of a subset of individuals with exceptionally high exercise tolerance, resulting in a markedly elevated ATHR and consequently leading to the observed proportional bias.

This study has some limitations. First, it did not consider specific types of β-blockers. It has been reported that αβ-blockers and β1-blockers have significantly different effects on HR responses during incremental exercise in patients with MI^23)^. Moreover, the current study did not address the dosage or duration of β-blocker administration, which remains a subject for future investigation. Second, in the multiple regression analysis, although both RHR and Hb were identified as statistically significant predictors of ΔHR, their regression coefficients were relatively low, indicating limited predictive power. Furthermore, the adjusted R^2^ value of the model was also small, suggesting that it explains only a small portion of the variability in ΔHR. This implies that other factors not assessed in this study, or those that cannot be objectively measured, may have a greater impact on ΔHR. Third, considering the reduced hospitalization periods with the widespread use of percutaneous coronary intervention, this study included patients who underwent CPX within 13 days, as recommended by the AT measurement guidelines. However, 11 patients who underwent CPX after ≥14 days were excluded. These patients may have experienced prolonged bed rest, making them more susceptible to orthostatic hypotension and tachycardia, which could influence HR. Such patients with severe AMI generally have low physical fitness and often require transfer to another hospital for convalescent rehabilitation rather than being discharged home. We therefore excluded these patients, as including them would have deviated from the study’s objective of focusing on those requiring accurate alternative exercise prescriptions during the short hospitalization period when CPX cannot be performed. Fourth, this study included only males. RHR^24)^ and the prevalence of older age^3)^ are generally known to be higher in females than in males; thus, whether the results are applicable to females remains unclear, and future studies should consider sex differences. The age at onset of AMI is generally higher in women^3)^, and continuous aerobic exercise at the AT level is often difficult due to frailty or musculoskeletal disorders. Similarly, CPX is frequently not feasible. Since most AMI cases occur in men^3)^, this study focused on male patients. In the future, the effectiveness of exercise therapy using a target HR of RHR + 25 in patients who meet the criteria of an RHR <91 bpm and Hb level ≥12.0 g/dL should be evaluated.

## Conclusions

This study is the first to demonstrate that individuals with a poor HR response during AT tend to have a higher RHR. In male patients with sinus rhythm who underwent early CPX after AMI, the RHR + 25 formula most closely approximated the ATHR. Furthermore, an RHR-based target HR setting can be most appropriately used when the RHR is <91 bpm and the Hb level is ≥12.0 g/dL, and is useful in situations where CPX cannot be performed.
